# Influence of Sucrose and Arenga pinnata Solutions on Enamel Surface Demineralization: A Profilometric Study

**DOI:** 10.7759/cureus.44592

**Published:** 2023-09-02

**Authors:** Annie Sylvea Valan, Jogikalmat Krithikadatta, Sashwat Sathish

**Affiliations:** 1 Dentistry, Saveetha Dental College and Hospitals, Saveetha Institute of Medical and Technical Sciences, Saveetha University, Chennai, IND; 2 Cariology, Saveetha Dental College and Hospitals, Saveetha Institute of Medical and Technical Sciences, Saveetha University, Chennai, IND; 3 Conservative Dentistry and Endodontics, Saveetha Dental College and Hospitals, Saveetha Institute of Medical and Technical Sciences, Saveetha University, Chennai, IND

**Keywords:** surface roughness, sucrose, stylus profilometer, palm sugar, demineralization

## Abstract

Background

Dental caries is a multifactorial disease that has the potential to impact individuals across various life stages. The influential role of sugar as a contributing risk element in the inception and advancement of dental caries is significantly pronounced.

Aim

The research aim was to analyze and compare the enamel surface roughness in teeth exposed to sucrose and *Arenga pinnata* (palm sugar) solutions by using a stylus profilometer

Materials and methods

In this investigation, 34 freshly extracted anterior teeth were obtained and they were split into two groups depending on the solution in which they were immersed. Group A consists of 17 teeth immersed in 1% sucrose solution supplemented in brain heart infusion (BHI) broth solution and Group B consists of 17 teeth immersed in 1% *Arenga pinnata *BHI broth. Each sample served as its own control. *Streptococcus mutans *was inoculated into these groups and they were immersed in their respective solution for five days. A stylus profilometer was utilized to measure the surface roughness of the teeth in this study. Data analysis involved paired t-tests for intragroup comparisons and independent t-tests for intergroup comparisons using SPSS software version 23.

Results

After five days of exposure to palm sugar or sucrose, it was observed that there was demineralization of the enamel surface on both samples. Although there was no statistical significance (p<0.05) when an independent t-test was conducted among these samples, there was a visible increase in the numerical values of Ra, Rq, Rz of teeth exposed to sucrose compared to palm sugar with a p-value of 0.529, 0.122 and 0.357, respectively.

Conclusion

From this study, it was concluded that although both sucrose and *Arenga pinnata* cause demineralization of enamel, it was shown that the latter caused lesser demineralization when compared to refined sugars to a certain extent. This study establishes a foundation for forthcoming investigations that could potentially explore the utilization of natural sugars as a substitute for sucrose, while also evaluating the mechanistic aspects underlying the impact of these sugars on enamel demineralization.

## Introduction

Dental caries is recognized as one of the most widespread diseases worldwide. It develops over time as a result of intricate interactions between carbohydrates, acid-producing bacteria, and host elements such as teeth and dental plaque. Numerous risk factors, such as poor oral hygiene, insufficient fluoride (F) exposure, decreased salivary flow, and excessive sugar consumption, among others, contribute to the development of dental caries [1,2].

Carbohydrate fermentation by oral bacteria, that are in an equilibrium state, initiates the process of demineralization. Consequently, there is an increase in the population of acidogenic and aciduric bacteria. Enamel and/or dentin that are exposed to the environment's constant acidity have their mineral equilibrium disrupted, which results in carious lesions [3,4]. The amount, frequency, and type of sugars consumed are three factors that are closely associated with the advancement of caries since studies have shown that people who often consume significant amounts of certain sugars have more severe caries than those who consume less of them [5,6].

Intracellular polysaccharides (IPS) contribute to the development of dental caries by extending the duration that the tooth surface is exposed to organic acids and preserving a pH level below the usual range in the plaque matrix. Insoluble extracellular polysaccharide (EPS) makes the plaque more porous and permits deeper sugar penetration into the biofilm, which could cause plaque catabolism and lower plaque pH values [7,8]. Moreover, entire biofilms created in the presence of sucrose have been discovered to include only trace amounts of minerals, inorganic phosphate (Pi), calcium (Ca), and F [9]. 

Previous studies have demonstrated a connection between sucrose consumption and insoluble EPS, as well as how these two factors play an important role in the development of dental caries [8]. Likewise, in situ research by Cury et al. showed that sucrose promoted greater enamel mineral loss than glucose and fructose did [10]. Thus, the preventative strategy for both public and private dental caries care has been recommended: consuming dietary sugar less frequently and substituting other sugars in foods [11]. Sugar substitutes are substances that the cariogenic organisms cannot break down, which prevents them from lowering the pH of the biofilm on the tooth surface. These substances are classified into two categories: bulk sweeteners (caloric), such as sorbitol, xylitol, and mannitol, and intense sweeteners (noncaloric), including aspartame, saccharin, sulfame, and glycyrrhizin.

These days, there are many different natural sweeteners available, including brown sugar, cane sugar, and palm sugar, each of which has a different nutritional profile. In Asia, palm sugar has been a common sweetener for thousands of years. They are used either directly or in formulation in sweet soy sauce, beverages, desserts, and a variety of traditional dishes. They are available in both granular and liquid form. Because it is natural, minimally processed, and nutritious, it is currently gaining appeal on a global scale. Its glycemic index (GI) is one of the main health benefits claimed [12]. Although widely cultivated in many regions of India, Sri Lanka, Myanmar, and Bangladesh, the Palmyra palm, which serves a variety of useful purposes, is thought to have originated in tropical Africa and is the source of palm sugar. Sucrose (70-80%), glucose (3-9%), and fructose (9-39%) make up the majority of palm sugar. It contains a lot of water, proteins, fat, Ca, and phosphorus [13].

However, in-depth studies about the effect of palm sugar in the formation of biofilm, dental caries initiation, and progression in comparison with sucrose have not been done. Hence this research aims to explore the effect of sucrose and *Arenga pinnata* solutions on enamel surface roughness measured as a function of surface demineralization. The objective of this study was to formulate sucrose and *Arenga pinnata* solutions, which were then exposed to the tooth enamel surface to assess its demineralization action in the presence of *Streptococcus mutans*. The hypothesis of this study was that there was a significant difference between the demineralization effects of *Arenga pinnata* solution and sucrose. The study's null hypothesis was that there was no difference between the demineralization effects of *Arenga pinnata* solution and sucrose.

## Materials and methods

This study was done after getting approval from the Institution’s Ethics Committee of Saveetha Dental College (IHEC/SDC/ENDO-2103/22/064). G*Power software 3.1.9.7 was used to calculate the sample size for this study, considering a previous study conducted by Tiwari et al. as a reference [14]. The total sample size obtained was 34 teeth (17 teeth in each group).

Preparation of tooth samples

**Figure 1:**
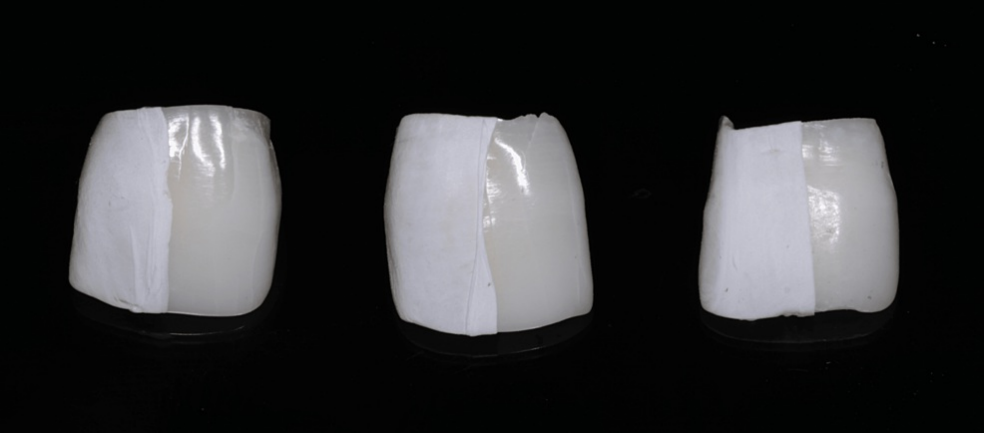
Demonstrates samples wrapped with Teflon tape

A total of 34 caries-free natural teeth that had been freshly extracted were collected from the tooth bank and included in this study. Using a diamond disk, the tooth crowns were separated from their roots, and they were subsequently stored in distilled water after which they were prepared for further analysis [15]. The samples were prepared on the buccal surface of the crown, ensuring that they were approximately 5×5 mm in size. Prior to analysis, all specimens underwent examination to identify any defects, such as white spots, cracks, or other issues that could potentially exclude them from the study. Every sample was wrapped with Teflon tape on one half of the tooth surface which served as the control as shown in Figure 1.

Inoculation of Streptococcus mutans

*Streptococcus mutans* culture utilized in this experiment was maintained in the microbiology department of Saveetha Dental College. The prepared samples (n=34) were divided equally into two different groups containing 17 samples each and designated as Group A (samples subjected to sucrose) and Group B (samples subjected to palm sugar). Each tooth sample from Group A and Group B was placed in a microcentrifuge tube, which was filled with 1 mL BHI medium (BHI broth) supplemented with 1% freshly prepared sucrose and 1% *Arenga pinnata* solutions, respectively. Fifty microliters of the overnight-grown *Streptococcus mutans* culture were added to all the samples and incubated at 37°C for 120 hours. Following the incubation period, the tooth samples were taken out from their respective solutions and rinsed thoroughly with deionized water [16].

Sample preparation for profilometric analysis

The samples were prepared adopting methodologies described by Fujii et al. in 2011 [17]. To enable the measurement of roughness parameters, circular molds with a diameter of 15 mm and a depth of 4 mm were utilized. Clear self-cured resin was used to fill the molds, and each tooth sample was then embedded in the resin with the labial surface of the teeth facing upward.

Measurement of surface roughness

**Figure 2:**
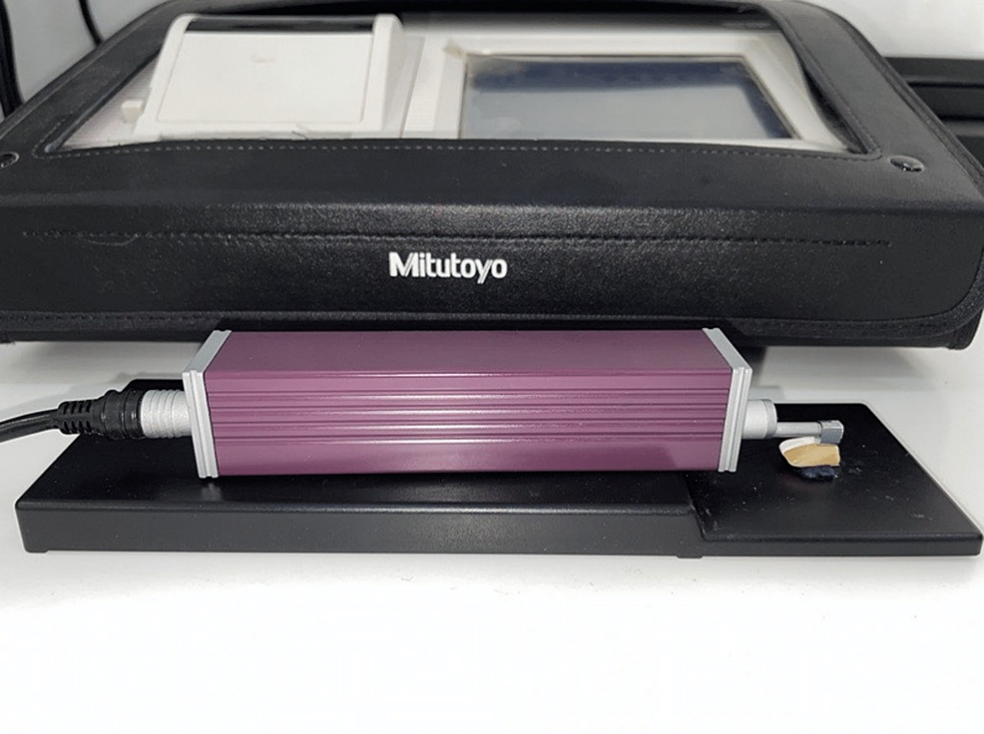
Demonstrates stylus profilometer, which is used to measure surface roughness

The surface roughness of teeth was measured with the help of a stylus profilometer, specifically the Mitutoyo Surftest SJ-310 model. The samples were placed perpendicular to the stylus tip as demonstrated in Figure 2 [18]. The stylus point was calibrated before use. The diamond tip of the stylus used in the profilometer had a radius of 2.00 μm. A testing force of 0.7 mN was applied during the measurement process. The diamond stylus moved at a speed of 0.25 mm/s, and the measuring line length for each sample was set to 5 mm [17,19]. The roughness of tooth samples was assessed using three parameters: Ra (roughness average), Rq (RMS roughness), and Rz (mean roughness depth). Ra is a measurement that represents the arithmetic average of the absolute values of the profile heights measured over the evaluation length. Rq, on the other hand, is the root mean square average of the profile heights observed over the evaluation length. Finally, Rz is the mean value of the five Rzi values obtained from the five sampling lengths calculated over the evaluation length. To minimize bias, the surface roughness measurements were conducted by a single operator.

Statistical analysis

SPSS Software (SPSS version 23.0, IBM, Chicago, USA) was used to carry out statistical analysis. To evaluate the assumptions of equality of variances and normal distribution of errors, the Shapiro-Wilk test was employed for all the variables. A paired t-test was employed to compare the roughness values before and after immersion in the respective solutions. Additionally, an independent t-test was conducted to compare the roughness induced by sucrose and palm sugar. A significance level (p-value) of 0.05 was set for all tests, indicating that any result with a p-value less than 0.05 was deemed statistically significant.

## Results

Table 1 represents a comparative analysis of Ra, Rq, and Rz values before and after subjecting teeth to immersion in palm sugar and sucrose solutions. The paired t-test was employed for this comparison. A significant difference was observed with respect to all three parameters before and after immersion in sucrose solution (p<0.05). These findings strongly indicate evident demineralization of the teeth following exposure to both palm sugar and sucrose solutions. 

**Table 1:** Comparison of Ra, Rq, and Rz values before and after immersion in palm sugar and sucrose solution using paired t-test

Group	Variable	Mean difference (S.D)	t	p
Sucrose	Ra	-1.0849 (0.49262)	12.842	0.000
Palm sugar	Ra	-1.18591 (0.47459)	14.570	0.000
Sucrose	Rq	-0.98185 (0.08556)	11.475	0.000
Palm sugar	Rq	-1.1090 (0.48120)	13.439	0.000
Sucrose	Rz	0.57618 (0.96505)	-3.481	0.001
Palm sugar	Rz	0.3500 (0.85962)	-2.374	0.024

**Figure 3:**
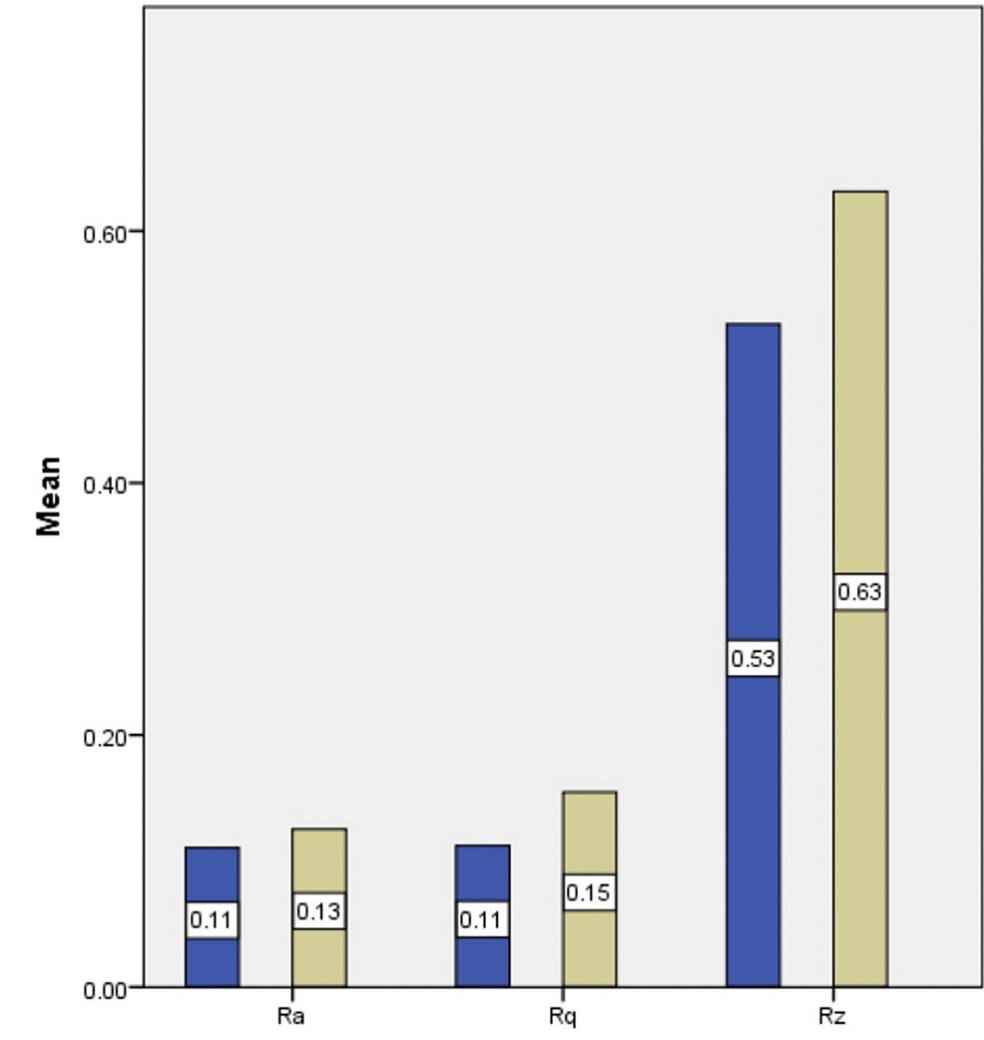
Mean Ra, Rq, Rz values of Group A and Group B (blue bar denotes palm sugar group and yellow bar denotes sucrose group) Ra, roughness average; Rq, RMS roughness; Rz, mean roughness depth

The comparison between two distinct groups: Group A and Group B, regarding their surface roughness measured through parameters Ra, Rq, and Rz values is mentioned in Table 1. It was observed that the average surface roughness values for Group A were higher than those for Group B, indicating that Group A surfaces tended to be rougher on average compared to Group B surfaces. The graphical representation of these findings can be found in Figure 3.

To determine the significance of these differences, statistical analysis was conducted by employing an independent t-test for this comparison and the results were presented in Table 2. Surprisingly, the statistical evaluation revealed that the disparities in surface roughness between the two groups were not statistically significant, with a p-value exceeding 0.05. Consequently, it was seen that there is no noteworthy variation in surface roughness between the two groups. 

**Table 2:** Comparison of Ra, Rq, and Rz values between palm sugar and sucrose using independent t-test Ra, roughness average; Rq, RMS roughness; Rz, mean roughness depth

Variable	Group	Mean (S.D)	t	p
Ra	Palm sugar	0.1105 (0.06508)	-0.636	0.529
Ra	Sucrose	0.1253 (0.07013)		
Rq	Palm sugar	0.1121 (0.07943)	-1.59	0.122
Rq	Sucrose	0.1546 (0.07646)		
Rz	Palm sugar	0.5262 (0.23041)	0.095	0.357
Rz	Sucrose	0.6313 (0.40271)		

## Discussion

The amount of sugar consumed worldwide is estimated to reach 198 million tonnes in 2027, with annual growth expected to be about 1.48% [20]. India is arguably the world's greatest user of sugar, and both availability and consumption have increased by three to four times. Free sugar being readily available and accessible for consumption on a daily basis, is a serious threat to health because it raises the chances of non-communicable diseases like diabetes, obesity, and dental cavities.

Numerous traditional sugars have been consumed on a regular basis in India for generations, including brown sugar, honey, jaggery, palm jaggery, and palm sugar. In order to ensure their children's optimum health, parents substitute these sugars in regular foods and beverages since they are seen to be a safer alternative to refined sugar [21]. Different physical forms of sugar have different demineralization and cariogenic potential. Demineralization leading to caries, being a dynamic process, could be influenced by frequency of consumption of sugars, time period of exposure, physical form, and type of tooth substrate. Hence, palm sugar was tested for cariogenicity in this study.

During an acidic attack or a normal demineralization process, both the organic and inorganic components of the tooth's matrix can undergo chemical dissolution. This phenomenon is brought on by the water content of dentine and enamel, which facilitates the diffusion of acid and mineral content out of teeth [22]. Studies have revealed that enamel becomes rougher after demineralization. This phenomenon was believed to be triggered by the surface precipitation of a new calcium phosphate phase [23,24].

Establishing a correlation between the dynamic caries process, which includes demineralization and remineralization, and objective quantitative data is of utmost importance. This correlation can be achieved by employing techniques such as profilometry for evaluating tactile sensations and reflectometry for visual assessment. The strategy used to evaluate surface roughness is crucial since the roughness value depends on the measuring method. Surface roughness can be assessed using various techniques and equipment, including Vickers diamond testing equipment, contact or stylus profilometers, non-contact optical profilometers, and scanning electron microscopes (SEM) [25]. A stylus is run across the surface by contact profilometers (CP) to record the surface profile. Even on samples with the most severe erosion, the depth of the CP-induced scratches was below 1 micrometer, making them unlikely to have an impact on the study's findings [26]. 

In this study, a CP was used to assess the surface roughness caused by sucrose and palm sugar. Sucrose had higher surface roughness values than palm sugar, with respect to all three parameters (Ra, Rq, Rz), although not significant. Previous studies have suggested that the cariogenicity of different sugars varies significantly [27]. Indeed, among various dietary sugars, sucrose has been commonly recognized as a major contributor to the development of caries and is often referred to as an "arch criminal" in this regard [28]. In early animal studies, it has been observed that feeding animals with different sugars at equal concentrations resulted in more severe caries lesions when sucrose was used compared to glucose, fructose, maize starch, and amylopectin [29,30]. Further investigation into the mechanisms underlying the cariogenicity of sucrose has revealed that it plays a significant role in the synthesis of water-insoluble glucans, which are major components of EPS [31]. Sucrose acts as the exclusive substrate for the production of these glucans, contributing to the formation of biofilms and dental plaque. Studies have also found that biofilms formed in the presence of sucrose have lower concentrations of Ca, phosphate (P), and F compared to those formed in the presence of glucose and fructose [11,32]. These elements serve as essential reservoirs of minerals that help prevent the demineralization of tooth hard tissues

In accordance with these findings, investigations employing an intraoral caries model indicated that *Streptococcus mutans* plaque made from cultures that contained sucrose had significantly more demineralization potential than plaque made from cultures that contained glucose [8,10]. Razdan et al. conducted a study where they observed that the mean depth of enamel demineralization was the lowest in the sucralose group, followed by honey, palm sugar, and glucose [33]. This was attributed to sucralose’s metabolically inert nature and its non‑cariogenicity in animal models.

Similarly, a study conducted to compare the enamel demineralization of honey, glucose, and fructose showed that glucose had the highest mean depth of demineralization, whereas honey had the least [34]. In a recent in vitro study utilizing a saliva-derived multispecies biofilm model, it was demonstrated that sucrose exhibited greater cariogenic potential compared to glucose and lactose as dietary sugars [35]. Clinical investigations are required to comprehensively understand the role of sucrose compared to other sugars and to evaluate current intervention strategies in clinical settings. Additionally, new approaches should be developed with the aim of thwarting sucrose’s cariogenic effects mediated by glucans. Promisingly, advancements in food science and the emerging concept of "functional foods" offer multiple avenues to reduce the cariogenic potential of high-sugar diets.

The present study does acknowledge several limitations that should be taken into consideration. First, the frequency and pH of the material and treatment substrate have not been taken into account. The duration of exposure to the palm sugar and sucrose solutions was only up to five days, which is relatively short. As such, future investigations incorporating long-term exposure durations are necessary to comprehensively understand the potential effects on dental health. Additionally, the study utilized planktonic bacteria, and further research exploring polymicrobial biofilm scenarios would be beneficial to offer a more comprehensive view of the actual oral environment. Moreover, it is crucial to highlight that this study was conducted in an artificial laboratory setting, which might restrict the generalizability of the findings to real-world clinical contexts. Therefore, caution should be exercised when extrapolating these results to actual clinical scenarios, and additional studies conducted under more realistic conditions are essential for a deeper understanding of the implications for dental health and disease prevention.

## Conclusions

This study has underlined the higher demineralization capacity of sucrose in comparison with palm sugar, as indicated by the changes in surface roughness. Nonetheless, given the absence of statistical significance between the groups, the null hypothesis has been substantiated. Additional in vivo studies are crucial for obtaining a comprehensive understanding of the intricate dynamics within the oral environment. These studies are necessary to explore various aspects, including the formation of complete dental biofilm and the pivotal role of saliva.

## References

[1] Usha C, Sathyanarayanan R (2009). Dental caries - a complete changeover (part I). J Conserv Dent.

[2] Xu Y, You Y, Yi L (2023). Dental plaque-inspired versatile nanosystem for caries prevention and tooth restoration. Bioact Mater.

[3] Arthur RA, Waeiss RA, Hara AT, Lippert F, Eckert GJ, Zero DT (2013). A defined-multispecies microbial model for studying enamel caries development. Caries Res.

[4] Gomar-Vercher S, Cabrera-Rubio R, Mira A, Montiel-Company JM, Almerich-Silla JM (2014). Relationship of children's salivary microbiota with their caries status: a pyrosequencing study. Clin Oral Investig.

[5] Holloway PJ, Moore WJ (1983). The role of sugar in the aetiology of dental caries. J Dent.

[6] Head D, Devine D, Marsh PD (2017). In silico modelling to differentiate the contribution of sugar frequency versus total amount in driving biofilm dysbiosis in dental caries. Sci Rep.

[7] Dibdin GH, Shellis RP (1988). Physical and biochemical studies of Streptococcus mutans sediments suggest new factors linking the cariogenicity of plaque with its extracellular polysaccharide content. J Dent Res.

[8] Zero DT, van Houte J, Russo J (1986). The intra-oral effect on enamel demineralization of extracellular matrix material synthesized from sucrose by Streptococcus mutans. J Dent Res.

[9] Ccahuana-Vásquez RA, Tabchoury CP, Tenuta LM, Del Bel Cury AA, Vale GC, Cury JA (2007). Effect of frequency of sucrose exposure on dental biofilm composition and enamel demineralization in the presence of fluoride. Caries Res.

[10] Cury JA, Rebelo MA, Del Bel Cury AA, Derbyshire MT, Tabchoury CP (2000). Biochemical composition and cariogenicity of dental plaque formed in the presence of sucrose or glucose and fructose. Caries Res.

[11] Moynihan P, Petersen PE (2004). Diet, nutrition and the prevention of dental diseases. Public Health Nutr.

[12] Maryani Y, Rochmat A, Khastini RO, Kurniawan T, Saraswati I (2021). Identification of macro elements (sucrose, glucose and fructose) and micro elements (metal minerals) in the products of palm sugar, coconut sugar and sugar cane. Adv Biol Res.

[13] Poeloengasih CD, Pranoto Y, Hayati SN (2016). A physicochemical study of sugar palm (Arenga pinnata) starch films plasticized by glycerol and sorbitol. AIP Conf Proc.

[14] Tiwari S, Saha S, Dhinsa K, Grover N, Gundewar MS, Tripathi AM (2022). Remineralizing potential of low-fluoridated, nonfluoridated and herbal nonfluoridated dentifrices on demineralized surface of primary teeth: an in vitro study. Int J Clin Pediatr Dent.

[15] Rajeev G, Lewis AJ, Srikant N (2020). A time based objective evaluation of the erosive effects of various beverages on enamel and cementum of deciduous and permanent teeth. J Clin Exp Dent.

[16] Cross SE, Kreth J, Wali RP, Sullivan R, Shi W, Gimzewski JK (2009). Evaluation of bacteria-induced enamel demineralization using optical profilometry. Dent Mater.

[17] Fujii M, Kitasako Y, Sadr A, Tagami J (2011). Roughness and pH changes of enamel surface induced by soft drinks in vitro-applications of stylus profilometry, focus variation 3D scanning microscopy and micro pH sensor. Dent Mater J.

[18] Barac R, Gasic J, Trutic N (2015). Erosive effect of different soft drinks on enamel surface in vitro: application of Stylus profilometry. Med Princ Pract.

[19] Gyurkovics M, Baumann T, Carvalho TS, Assunção CM, Lussi A (2017). In vitro evaluation of modified surface microhardness measurement, focus variation 3D microscopy and contact stylus profilometry to assess enamel surface loss after erosive-abrasive challenges. PLoS One.

[20] (2023). OECD‐FAO Agricultural Outlook 2018‐2027. https://www.agri-outlook.org/Agricultural-Outlook-2018.pdf.

[21] Srudhy R, Nijesh JE, Chaly PE (2020). Effect of various sweeteners on cariogenicity features of Streptococcus mutans: In-vitro study. Med Legal Update.

[22] Shellis RP, Featherstone JD, Lussi A (2014). Understanding the chemistry of dental erosion. Monogr Oral Sci.

[23] Groenhuis RA, Jongebloed WL, ten Bosch JJ (1980). Surface roughness of acid-etched and demineralized bovine enamel measured by a laser speckle method. Caries Res.

[24] Abou Neel EA, Aljabo A, Strange A (2016). Demineralization-remineralization dynamics in teeth and bone. Int J Nanomedicine.

[25] Ersahan S, Alakus Sabuncuoglu F (2016). Effect of surface treatment on enamel surface roughness. J Istanb Univ Fac Dent.

[26] Heurich E, Beyer M, Jandt KD, Reichert J, Herold V, Schnabelrauch M, Sigusch BW (2010). Quantification of dental erosion - a comparison of stylus profilometry and confocal laser scanning microscopy (CLSM). Dent Mater.

[27] Horton WA, Jacob AE, Green RM, Hillier VF, Drucker DB (1985). The cariogenicity of sucrose, glucose and maize starch in gnotobiotic rats mono-infected with strains of the bacteria Streptococcus mutans, Streptococcus salivarius and Streptococcus milleri. Arch Oral Biol.

[28] Newbrun E (1969). Sucrose, the arch criminal of dental caries. ASDC J Dent Child.

[29] Grenby TH, Hutchinson JB (1969). The effects of diets containing sucrose, glucose or fructose on experimental dental caries in two strains of rats. Arch Oral Biol.

[30] Frostell G, Keyes PH, Larson RH (1967). Effect of various sugars and sugar substitutes on dental caries in hamsters and rats. J Nutr.

[31] Paes Leme AF, Koo H, Bellato CM, Bedi G, Cury JA (2006). The role of sucrose in cariogenic dental biofilm formation - new insight. J Dent Res.

[32] Tenuta LM, Del Bel Cury AA, Bortolin MC, Vogel GL, Cury JA (2006). Ca, Pi, and F in the fluid of biofilm formed under sucrose. J Dent Res.

[33] Razdan TR, Singh VP, Rav AB, Harihara M, Sreeram SR (2016). Comparative evaluation of enamel demineralization depth by five sweeteners: an in-vitro study. J Int Oral Health.

[34] Ahmadi-Motamayel F, Rezaei-Soufi L, Kiani L, Alikhani MY, Poorolajal J, Moghadam M (2013). Effects of honey, glucose, and fructose on the enamel demineralization depth. J Dent Sci.

[35] Du Q, Fu M, Zhou Y (2020). Sucrose promotes caries progression by disrupting the microecological balance in oral biofilms: an in vitro study. Sci Rep.

